# An Updated Understanding of the Role of YAP in Driving Oncogenic Responses

**DOI:** 10.3390/cancers13123100

**Published:** 2021-06-21

**Authors:** Giampaolo Morciano, Bianca Vezzani, Sonia Missiroli, Caterina Boncompagni, Paolo Pinton, Carlotta Giorgi

**Affiliations:** Laboratory for Technologies of Advanced Therapies (LTTA), Section of Experimental Medicine, Department of Medical Science, University of Ferrara, 44121 Ferrara, Italy; mrcgpl@unife.it (G.M.); bianca.vezzani@unife.it (B.V.); sonia.missiroli@unife.it (S.M.); caterina.boncompagni@unife.it (C.B.); paolo.pinton@unife.it (P.P.)

**Keywords:** cancer, YAP, Hippo pathway, targeted therapies, immunity

## Abstract

**Simple Summary:**

In 2020, the global cancer database GLOBOCAN estimated 19.3 million new cancer cases worldwide. The discovery of targeted therapies may help prognosis and outcome of the patients affected, but the understanding of the plethora of highly interconnected pathways that modulate cell transformation, proliferation, invasion, migration and survival remains an ambitious goal. Here we propose an updated state of the art of YAP as the key protein driving oncogenic response via promoting all those steps at multiple levels. Of interest, the role of YAP in immunosuppression is a field of evolving research and growing interest and this summary about the current pharmacological therapies impacting YAP serves as starting point for future studies.

**Abstract:**

Yes-associated protein (YAP) has emerged as a key component in cancer signaling and is considered a potent oncogene. As such, nuclear YAP participates in complex and only partially understood molecular cascades that are responsible for the oncogenic response by regulating multiple processes, including cell transformation, tumor growth, migration, and metastasis, and by acting as an important mediator of immune and cancer cell interactions. YAP is finely regulated at multiple levels, and its localization in cells in terms of cytoplasm–nucleus shuttling (and vice versa) sheds light on interesting novel anticancer treatment opportunities and putative unconventional functions of the protein when retained in the cytosol. This review aims to summarize and present the state of the art knowledge about the role of YAP in cancer signaling, first focusing on how YAP differs from WW domain-containing transcription regulator 1 (WWTR1, also named as TAZ) and which upstream factors regulate it; then, this review focuses on the role of YAP in different cancer stages and in the crosstalk between immune and cancer cells as well as growing translational strategies derived from its inhibitory and synergistic effects with existing chemo-, immuno- and radiotherapies.

## 1. The Hippo Pathway Core

The Hippo signaling pathway probably does not affect all functions that are currently known to be carried out under physiological and disease conditions. Indeed, Hippo refers to the serine/threonine protein kinase Hpo, originally identified in *Drosophila melanogaster* to produce a “hippopotamus”-like phenotype as a consequence of tissue overgrowth [1,2,3,4]. In this review, we will describe some aspects of this pathway considering the mammalian orthologous proteins and considering the similarity in function in cancer environments.

**Figure 1:**
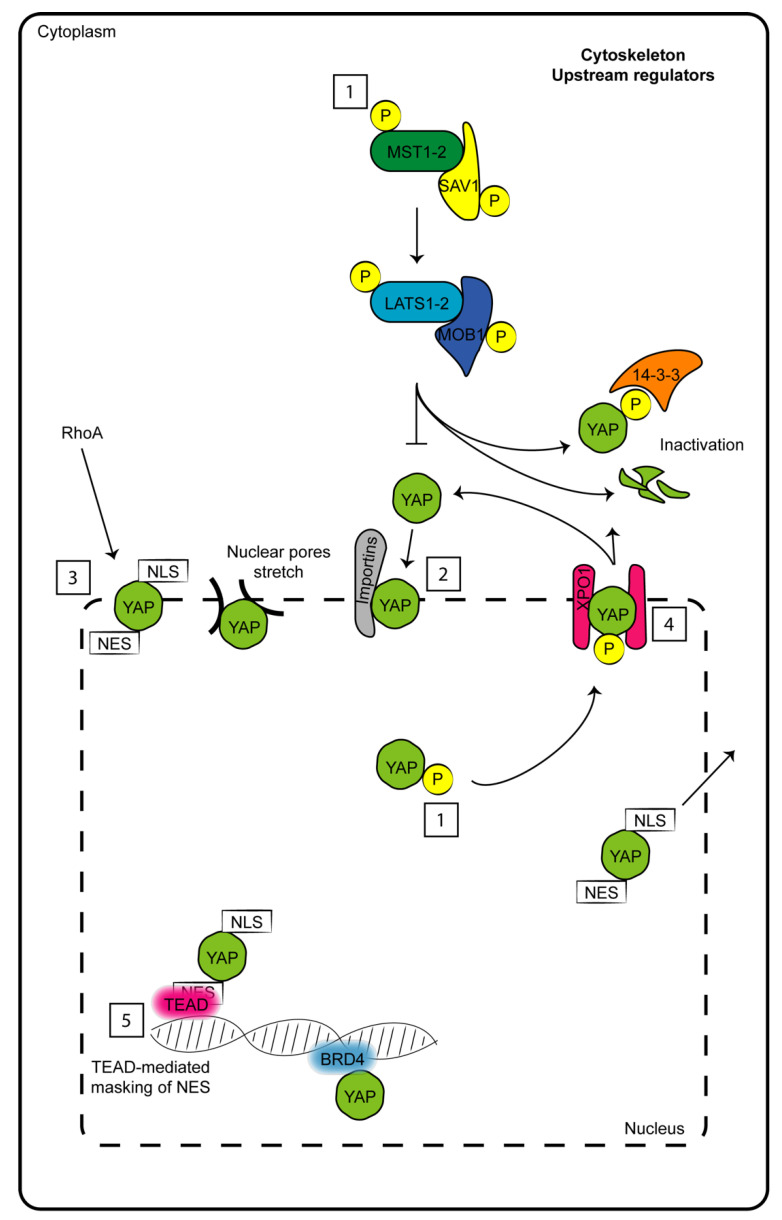
Schematic representation of factors which determine to date nuclear–cytoplasm shuttling of YAP. (1) Phosphorylations and dephosphorylations of both upstream Hippo members and YAP. (2) Nuclear import mediated by importins. (3) Nuclear import through nuclear localization sequence (NLS) and mediated by RhoA. (4) Nuclear export mediated by phosphorylations, nuclear exporting sequence (NES) and exportin 1 (XPO1). (5) Nuclear restraint caused by masking NES motif by TEAD or other partner interactions.

The key components of the Hippo pathway may be included in two complex modules: the first module is based on serine-threonine kinase activity, and the second module involves co-transcriptional effectors. Proteins with kinase activities are mammalian Ste20-like (MST) kinases 1-2 and large tumor suppressors (LATSs) 1-2. On the other hand, the transcriptional coactivators are represented by Yes-associated protein (YAP) 1 and WW domain-containing transcription regulator 1 (WWTR1, also named TAZ) [1]. A cascade of multiple phosphorylation events (at multiple sites) occurs from MST1-2 to YAP. MST1-2 heterodimerizes with Salvador (SAV) 1 through the sav/Rassf/Hpo (SARAH) domain, which allows the phosphorylation of LATS1-2 but also of SAV1 and MOB1. LATS1-2 phosphorylates YAP and TAZ by binding to its partner MOB1. When the kinase module is inactive, YAP and TAZ are not phosphorylated and translocate from the cytosol to the nucleus, where they induce the expression of several genes involved in antiapoptotic processes by targeting members of the TEA domain (TEAD) transcription factor family. When MST1-2 and LATS1-2 are active and phosphorylated, they mediate the phosphorylation of YAP and TAZ, which remain anchored to 14-3-3 in the cytosol and may be degraded by the proteasome [5] (Figure 1). Despite these cycles of phosphorylations and dephosphorylations have constituted the main regulator mechanisms for YAP shuttling from the cytoplasm to the nucleus for years, the state of the art of these mechanisms shouldn’t be that simple. Indeed, ten years ago, a study by Wada et al. reported a significant YAP restrain into the nucleus in spite of its phosphorylation by LATS1-2 [6]. Moreover, in the last four years, three studies provided additional insights on the “rules” governing this mechanism of nucleo-cytoplasm shuttling, which constitutes as druggable for therapeutic strategies against cancer. Basically, the shuttling of YAP is continuous and at the equilibrium inside cells at “resting conditions”. This equilibrium can be impaired under different stimulations through the action of upstream pathways (reported in chapter 2) that intersect with YAP. For example, stimuli that induce LATS-mediated phosphorylation of YAP, increase its export rate from the nucleus through the adaptor protein exportin 1 (XPO1) [7] (Figure 1).

Manning et al. provided evidence that dynamic fluctuations of YAP localization are cell-type- and cell-cycle-dependent [8]. YAP function would be dependent to the rate of its nuclear import which, in turn, depends exclusively on Hippo proteins and mediate YAP interactions with Importin α1 [8,9]. Interestingly, despite YAP and TAZ do not have a nuclear localization sequence, Kofler M. et al. for the first time unveiled a non-canonical motif in their sequences which ensure their movement across the nuclear envelope. Noteworthy, its nuclear export sequence would overlap with the position to which TEAD partners interact with the co-transcriptional factors, thus making this site useful for nuclear export [10] (Figure 1).

Currently, all these studies are not sufficient to definitively outline which is the preferred “force” driving YAP localization. To date, a combination of induced nuclear import/export sequences, interaction with proteins at the nuclear envelope or close to the DNA and post-translational modifications regulate nuclear YAP accumulation in a mode which is strongly dependent from cell-type and external stimulus (Figure 1).

This apparently simple pathway becomes complicated if several points that will be outlined below are considered, or if the countless upstream regulators, the complex crosstalk with multiple additional pathways acting in parallel, the integration of signals with tissue specificity and the continued discovery of new players are considered [11].

### Structural and Functional Differences between YAP and TAZ

The structural differences between YAP and TAZ are minimal. YAP is a 488-amino-acid protein that is composed of an amino-terminus (N-terminus) rich in prolines; a TEAD-binding domain (BD) that constitutes a platform interaction site for multiple TEAD factors [12]; one 14-3-3 BD, which is able to interact with 14-3-3 family proteins once YAP becomes phosphorylated and is responsible for its cytoplasmic retention; two WW domains (so-called for the presence of two highly conserved tryptophan residues) that recognize a series of two prolines and one tyrosine separated by any amino acid (PPXY), which is a domain found in many transcription factors; one transcriptional activation domain (TAD); and one PDZ BD at the carboxyl terminus (C-terminus), which regulates the nuclear localization and is a binding site for additional proteins carrying PDZ domains [5,13]. From this concise description, it is clear that the protein exerts all its functions entirely by binding multiple partners; indeed, it does not have the possibility to interact directly with DNA [14].

**Figure 2:**
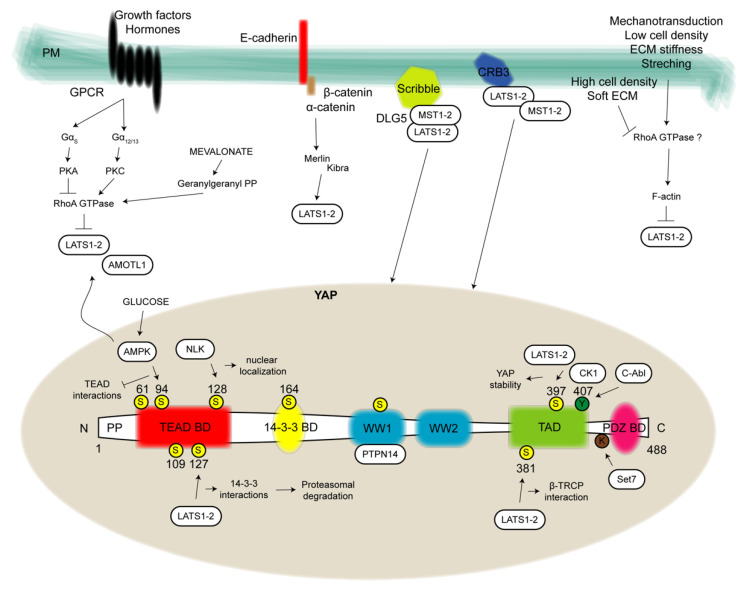
YAP structure and regulation. In the present figure, YAP structure (bottom) is depicted by showing all binding domains allowing protein-protein interactions to exert YAP pro-tumorigenic functions. From the N-terminus to C-terminus of the 488 aa protein it can be distinguished a proline-rich domain (PP), one TEAD binding domain (BD), one 14-3-3 BD, two WW domains, one transcriptional activation domain (TAD) and one PDZ BD. Yellow circles highlight phosphorylation sites at serine (S). Green circles evidence phosphorylation sites at tyrosine (Y) and one additional site for lysine (K) methylation is reported. Close to these regulatory sites, kinases or methyltransferase are represented in white boxes and the pathway that in turn they regulate. Upstream modulators (top) are depicted in correspondence of the plasma membrane (PM). In detail, on the left G-protein-coupled receptors (GPCRs)-mediated signal transduction by which several growth factors and hormones regulate the kinase module of the Hippo pathway via RhoA-GTPases. Going forward, the cadherin-catenin complex involved in both structural roles and in the cytoplasmic restrain of YAP. YAP cytosol-nucleus shuttling is also modulated by proteins involved in cell polarity and tight junctions, such as the Scribble complex, Crumbs (CRB) family members and Merlin which mainly operate for the recruitment, stabilization and function of the kinase module. On the right, signals of mechanotransduction such as cell density, extracellular matrix (ECM) stiffness and stretching are reported. AMOTL1: angiomotin-like 1; AMPK: AMP-activated protein kinase; C-Abl: Abl proto-oncogene; CK1: casein kinase 1; DLG5: disc large 5; NLK: nemo-like kinase; PKA: cAMP-dependent protein kinase; PKC: protein kinase C; PTPN14: nonreceptor tyrosine phosphatase 14; Set7: SET-domain containing 7, histone lysine methyltransferase.

On the other hand, TAZ is a 400-amino-acid protein with only one WW domain, four (instead of five) serine residues that are the target of LATS1 and two activities, one site for phosphorylation by casein kinase (CK) 1 and no additional regulatory sites included in the YAP structure [13] (Figure 2).

Notably, among all phosphorylation sites, the two most important for YAP inhibition are the serines at positions 127 (S127) and S381, while for TAZ, the two most important sites are S89 and S311 [15].

The first functional difference between the co-transcriptional factors lies in their appearance during embryo development. YAP is translated in early stages (E3.5, blastocyst); however, TAZ is not detectable before stage E6.5. This difference might, in principle, explain the observation that the phenotype resulting from the loss of YAP during embryonic development cannot be compensated by the functions of TAZ [16] because YAP is absent. Nevertheless, YAP KO and wild-type (WT) phenotypes become significantly different from each other between stages E8.5 and 10.5 [16].

The loss of TAZ is reported to increase the expression and resulting function of YAP in nearly all stages of life. For example, in a model of human corneal stroma fibrosis, in which transforming growth factor beta (TGF-β) transforms keratocytes in myofibroblasts by activating fibroblasts in a Hippo-dependent manner, the presence of TAZ limits YAP function; otherwise, the downregulation of YAP, which does not interfere with TAZ expression, counteracts the fibrosis process [17]. TGF-β pathway and YAP/TAZ signaling are one of many examples of how the latter is highly interconnected to several molecular routes inside cells at multiple levels. TGF-β signaling is prone to regulate either proliferation or apoptosis, thus covering a dual role in cancer [18]. One factor of regulation between both pathways is the subcellular localization of their players. Small mothers against decapentaplegic (SMAD) proteins (which are in turn subdivided in regulators, coactivators and inhibitors) are key transducers for receptors of TGF-β and move towards the nucleus from the cytoplasm and vice versa [19], as YAP/TAZ did. In this scenario, it has been reported that once into the cytoplasm YAP may interact with SMAD7 (inhibitory SMAD) to promote the repression of the SMAD- and TGF-β-mediated signaling [20]. Otherwise, into the nucleus the complex formation between YAP/TAZ and regulatory and coactivator SMAD (2,3,4 isoforms) triggers strong transcriptional activities [21] critical in wound healing.

Some reports described a relevant function of YAP compared to TAZ [22,23], and this function was highlighted by developmental studies in embryos lacking YAP. YAP-KO embryos become impaired by several defects in vasculogenesis and chorioallantoid fusion in addition to their small size [24]. Here, the presence or modulation of TAZ does not improve this condition.

Moreover, YAP, when induced, triggers proteosomal-dependent degradation of TAZ [25] through its TAD and PDZ motifs and via the crosstalk of the GSK-3β-dependent phosphorylation cascade and the β-catenin pathway [26]. This process is unidirectional and finely regulates TAZ abundance in mammalian cells [25]. Although in contrast with what was described above, this regulatory mechanism, if confirmed with additional mechanistic insights and in other experimental settings, would at least partially explain a couple of observed biological events: the high turnover of TAZ (compared to the stability of YAP and considered the “double” regulation of the protein by ubiquitin on the additional phosphodegron at the N-terminus not shared by YAP) and the tumor suppressor properties of YAP [27,28]. Indeed, TAZ downregulation promotes drastic cell death.

To complicate the matter, these proteins may also differ in a tissue-specific way. Interactions between YAP/TAZ and TEAD factors constitute another point of nonredundancy of the proteins. The determination of the crystal structure of the TAZ-TEAD heterodimer revealed the possibility that TAZ binds tandemly located TEAD factors efficaciously; this mode would differ from the mode used by YAP to form 1:1 heterodimer complexes [29]. This difference might suggest differential activation of downstream genes, as the recently identified angiomotin-like 2 (AMOTL2) and FOS-like 1 (FOSL1) genes are dependent on only one effector rather than the other effector [16].

TEADs are characterized by an N-terminus sequence deputed to bind DNA and a C-terminus representing the transactivation domain involved in YAP binding. YAP encircles TEADs with its N-terminus portion and gives rise to three interfaces composed by B-sheets and helices. The third interface assumes importance because it determines the affinity of binding between molecular partners, while the second interface is home of peptides and small molecules bindings that may act as disruptor of YAP/TEAD interaction. These works are carried out by Hong et al. and Pobbati et al. groups who recently identified another pocket, showed as target for drugs with great potential in the impairment of YAP/TEAD complex formation [30,31].

From all these findings, including the fact that double KO of YAP/TAZ leads to stronger biological effects than simple YAP absence [32], YAP and TAZ do not always have redundant roles, and the role of TAZ should be revised and better elucidated, although YAP is currently considered the “predominant twin” and shows growing attention as the main target for new therapeutic strategies in cancer diseases.

## 2. Extracellular and Intracellular Modulators of YAP

YAP undergoes constant and rapid phosphorylation and dephosphorylation in response to a multitude of intrinsic and extrinsic signals but not always in a Hippo-dependent manner [5]. These multiple signaling cascades may act in parallel, cooperate to reach stronger responses, or contrast with each other [32].

Overall, we can mention at least four large groups of upstream pathways that regulate YAP nuclear localization and function [33], subdivided into: (i) mechanotransduction; (ii) polarity and tight junction proteins; (iii) the cadherin-catenin complex; and (iv) soluble growth factors.

### 2.1. Mechanotransduction

YAP is considered a potent sensor for mechanical stimuli that links communication signals from the external milieu, such as those generated by cell density, extracellular matrix (ECM) morphology and stiffness of substrates [32], to several nuclear-derived functions. Thus, YAP is at the center of a feedback regulatory mechanism by which a cell regulates the shape and tension of the F-actin cytoskeleton on the basis of the surrounding environment.

Homophilic cell-cell contacts in confluent cells inhibit cell growth through signals mediated by the cytoplasmic localization of YAP. Otherwise, under low cell density conditions, YAP predominantly localizes in the nucleus, where it activates TEAD-mediated co-transcription [34,35].

Cell shape and cytoskeletal organization are closely related to the ability of the cell to attach to the ECM, which provides structural support. As a result, YAP responds to changes that take place at the level of the whole tissue. Cells cultured on stiff ECM show high levels of nuclear YAP, whereas YAP is inhibited and localized in the cytoplasm in an soft ECM environment [36]. ECM stiffness and cell shape have a strong impact on the architecture and properties of the actin cytoskeleton [32,37]. Two different groups reported contrasting results regarding modulation of the Hippo pathway by mechanical and physical cues. DuPont revealed that cell geometry and F-actin regulate YAP in a Hippo-independent manner. This mechanism would require Rho GTPases and a given tension of the cytoskeleton, maintaining distance of these Rho GTPases from LATS1-2, which are the main upstream mediators of YAP [38]. Wada et al. proposed that LATS1-2 is essential for the function of F-actin in the regulation of YAP. Under conditions of low cell densities, cells are flat and spread, and this status promotes the formation of F-actin filaments. F-actin filaments inhibit LATS1-2 and, consequently, induce YAP dephosphorylation and nuclear localization, promoting cell proliferation. Otherwise, at high cell densities, cells become round, and F-actin filaments are less prominent and activate LATS1-2 [6]. A feedforward model may be activated: loss of F-actin filaments in a high confluence condition further triggers LATS1-2 kinase and keeps YAP in its inactive status, while abundant filaments induce LATS1-2 inactivation and maintain nuclear localization of YAP.

When cells undergo malignant transformation, this loop is deregulated, and F-actin filaments are lost, impairing autophagosome formation [39]. Moreover, F-actin inhibits tight junction binding proteins and AMOTs, promoting YAP translocation [40]. Angiomotin family proteins, included as Hippo components in the last 10 years, directly interact with YAP; some studies suggest a YAP-inhibitory function of these proteins, increasing its localization to tight junctions and its consequent phosphorylation [41], while other independent groups reported AMOTs as being required for YAP functions with nuclear maintenance [42].

The dual role of AMOTs reported by the studies could be due to post-translational modifications and thus to either their hyper- or hypophosphorylation status [43]. A correlation among cell density, cell geometry and stress fiber quantity in YAP control is evident. Different reports have identified MAP4K family members as an alternative kinase pathway able to directly phosphorylate LATS1-2. Interestingly, in response to different signals such as contact inhibition and F-actin disassembly, the silencing of both MAP4Ks and MST1-2 is required to suppress YAP phosphorylation [44,45]; indeed, different kinases may preferentially act to activate LATS1-2 depending on the upstream signals.

Cells are physically connected to the ECM through focal adhesions, which detect external mechanical signals and translate them into biochemical information through integrin-related pathways. The β1-integrin pathway, which involves focal adhesion kinase Src, is activated by stiff ECM and facilitates YAP nuclear localization by affecting LATS1-2 activity [46,47]. Src inhibitors can rapidly block the nuclear translocation of YAP under serum starvation conditions and in cells under conditions of low confluence. Deletion of LATS1-2 abolishes the effects of the inhibitors, indicating that Src-mediated regulation of YAP depends on LATS1-2. This topic will be further explained in the following sections.

The nucleus itself might be considered a mechanotransducer for YAP regulation; indeed, forces applied to the nucleus (very likely through the cytoskeleton) are sufficient to represent a signal for YAP nuclear translocation and for the reduction of the mechanical resistance of pores localized on its membrane [48] (Figure 1).

### 2.2. Polarity and Tight Junction Proteins

Several studies have demonstrated that either the deregulation or the loss of components of cell-cell junctions and cell polarity are permissive steps in cancer cell transformation and metastasis [49]; other evidence suggests concomitant silencing of the Hippo pathway and consequent YAP overactivation with nuclear localization [50]. Three major protein complexes control apical-basolateral polarity.

The first of these protein complexes is Scribble, whose plasma membrane (PM) localization is required for correct cell polarity [51], and the cytoplasmic retention of YAP is favored by direct action on the kinase module (both MST and LATS). Loss of Scribble leads to a significant nuclear localization of YAP, which interferes with the role of kinases in its phosphorylation. Scribble forms a complex with two additional proteins named lethal giant larvae (Lgl) and discs large (Dlg); recently, DLG5 was also identified as a scaffold for MST1-2 [52]. DLG5 KO mice show defects in apical-basal polarity; here, DLG5 acts as a negative regulator of MST1-2 and the Hippo pathway. DLG5 also stably interacts with microtubule affinity-regulating kinase 3 (MARK3) proteins and prevents binding between LATS1-2 and MST1-2, turning attention to a possible mechanism of YAP regulation via MST1-2 inactivation [53].

The second important regulator of cell polarity is the Crumbs (Crb) family. One of its members, CRB3, is associated with tight junctions and involved in cell–cell contact inhibition; interestingly, its downregulation leads to an increase in nuclear YAP [54]. Indeed, when CRB3 is expressed, it interacts with LATS1-2 and is responsible for the correct phosphorylation of YAP, as observed in studies on mice [55]. An additional level of YAP activity regulation is mediated by nonreceptor tyrosine phosphatase 14 (PTPN14) by directly binding to the WW domain of YAP and suppressing its activity [56]. Conversely, depletion of PTPN14 promotes the nuclear localization of YAP and improves cancer cell migration. However, other molecular mechanisms of Crumbs remain unclear.

The last modulator of YAP signaling involved in cell polarity is Merlin (encoded by a tumor suppressor gene, NF2) and its adaptor protein Na^+^/H^+^ exchanger regulatory factor (NHERF); Merlin is localized in the inner portion of the PM and constitutes a bridge linking adherens junctions to the kinase module of the Hippo pathway, and it is able to recruit and stabilize LATS1-2. Thus, Merlin is a strong inhibitor of YAP [57]; however, its loss triggers the impairment of adherens junctions and tissue overgrowth due to YAP nuclear localization. Adherens and tight junctions are increased in confluent cells and contribute to LATS1-2 activation and YAP silencing [35,58].

### 2.3. The Cadherin-Catenin Complex

Cadherins are a family of proteins that play a structural role on the surface of neighboring cells in solid tissues [59]. Different types of cadherins (in this paragraph, we refer to the Epithelial, E) exist among tissues, and with their multiple, transmembrane and calcium-dependent domains, they participate in cell adhesion [60], playing a pivotal role in maintaining a mechanical balance. E-cadherins form a conserved complex with β-catenin and can mediate several signaling pathways relevant to cancer, such as epithelial-mesenchymal transition (EMT) [61,62,63], fibrosis [64] and oncogene activation [65].

Loss of E-cadherin-β-catenin complexes directly controls the nuclear localization of YAP in normal and transformed cells [66,67], which promotes cell proliferation but also inhibits organogenesis. The finding that the MDA-MB-231 cell line, which lacks E-cadherin, exhibits nuclear localization of YAP even under high cell density conditions further supports the role of E-cadherin in cell density-mediated regulation of YAP [67]. Direct and indirect mechanisms have been hypothesized: from the concomitant recruitment of 14-3-3 and the sequestration of YAP to the junctions [68], to the stabilization of the kinase module function [67] or to Akt activation and Src inhibition [69].

Merlin also directly associates with α-catenin to promote adherens junction formation [70]. α-Catenin depletion induces the delocalization of Merlin from the junctions and leads to an increase in nuclear YAP, suggesting that α-catenin may regulate YAP through different mechanisms [46]. Proliferation assay experiments showed that Merlin and NHERF are necessary for the inhibition of proliferation mediated by E-cadherin [67].

### 2.4. Soluble Growth Factors

In addition to the previously mentioned upstream regulators, YAP can be further regulated by extracellular signals, which include growth, metabolic and inflammatory factors and hormones. These signals preferably operate as ligands of different classes of G-protein-coupled receptors (GPCRs) that transduce signals into the cytoplasm of cells. Indeed, GPCRs, the largest class of cell surface receptors, are activated in response to specific soluble factors and, depending on the types of coupled G protein, are able to induce or repress YAP functions [71,72,73]. The multitude of agonist–receptor complexes suggests that the regulation of YAP via GPCRs can be quite complex: the outcome may depend on the cell type, which receptor is expressed, and which G protein is coupled to the receptor.

#### 2.4.1. Growth Factors

The Hippo pathway is inhibited by several growth factors; the most common are epidermal growth factor (EGF), lysophosphatidic acid (LPA) and insulin-like growth factor (IGF) [74,75]. The main action of these factors is the activation of YAP target genes by mediating their massive translocation into the nucleus. This translocation is strongly linked to YAP-mediated cell proliferation and to as the importance of the mitogenic signals. Few studies have been reported about this topic, and the available studies seem to suggest that this phenomenon is mediated by the phosphatidylinositol 3-kinase (PI3K)-3-phosphoinositide-dependent protein kinase 1 (PDPK1) axis but not by AKT signaling [71]. Following this pathway, growth factors stimulate PI3K, which severely impairs the ability of LATS1-2 to phosphorylate YAP.

#### 2.4.2. Hormones

Hormones, such as glucagon and epinephrine, induce Gαs-coupled GPCR signaling and lead to the silencing of YAP through the cAMP/PKA-dependent inhibition of RhoA GTPase [73]. Park et al. provided genetic and biochemical evidence that supports the involvement of Gα 12/13 and its role in the alternative Wnt-YAP axis. These authors characterized alternative Wnt-YAP signaling involving LATS1-2 activation mediated by Gα 12/13—Rho GTPases to promote YAP activation [76].

#### 2.4.3. Metabolic Factors

Glucose deprivation in culture medium and the inhibition of glycolysis increase YAP phosphorylation and thus its inactivation, leading to it being retained in the cytoplasm [77]. AMP-activated protein kinase (AMPK), a cellular energy sensor that is sensitive to glucose, can directly phosphorylate YAP and interact with AMOTL1 and, in turn, LATS1-2 [78]. These observations suggested that AMPK is involved in both the direct and indirect regulation of YAP. Notably, LATS1-2 was also activated under AMPK KO conditions, indicating that cellular energy stress might activate additional AMPK-independent pathways [58,79]. Associated with this issue, YAP has been found to be directly regulated by the hexosamine biosynthesis pathway (HBP) in response to glucose availability. O-GlcNacylation of S109 YAP disrupts its interaction with LATS1-2, preventing its phosphorylation and translocation into the nucleus [80]. In addition, Enzo et al. observed that phosphofructokinase (PFK1), which is responsible for the limiting step of glycolysis, induces the interaction between YAP and TEAD [77].

Another metabolic mechanism implicated in YAP regulation is the mevalonate pathway, which is essential for synthesizing bioactive molecules, such as cholesterol, bile acid and isoprenyl derivatives. Geranylgeranyl pyrophostate is required for the activation of Rho GTPases, which can inhibit the phosphorylation of YAP and promote its nuclear localization. Additionally, in cancer cell lines, the activation of YAP activity was correlated with the increase in mevalonic acid induced by transcriptional activity of sterol regulatory element-binding proteins (SREBPs). The simultaneous depletion of SREBP1 and SREBP2 in vitro reduced the mevalonate-dependent nuclear localization of YAP to induce a significant decrease in YAP target gene expression [81]. In cancer, high levels of mevalonic acid due to strong activation of the SREBP transcriptional activity promote YAP nuclear translocation. Consistent with this activation, the mevalonate-YAP axis is central in cancer cell proliferation and self-renewal [81].

Hypoxia modulates Hippo signaling through the degradation of LATS2 through a process mediated by the ubiquitin ligase SIAH2 [82]. This mechanism leads to the activation of YAP and is critical for promoting the growth of breast cancer cells. YAP was also found to be activated in hepatocellular carcinoma cells under hypoxic conditions and in hypoxic regions in tissue models, underlying the importance of YAP in the hypoxic microenvironment of solid tumors [83]. Crosstalk between osmotic pressure and Hippo signaling was also proposed. Osmotic stress induces YAP nuclear localization in a LATS-independent manner. Osmotic stress works via a mitogen-activated protein kinase, NLK, which induces YAP phosphorylation at the S128 residue. The phosphorylation of S128 influences the ability of YAP to bind to 14-3-3, even if S127 is phosphorylated by LATS, inducing YAP nuclear accumulation [84].

In summary, it is reasonable to suppose that YAP represents a complex cellular system where multiple regulators of the Hippo pathway and YAP have been identified, but most of these regulators remain to be elucidated.

## 3. YAP-Dependent Signaling Pathways Impacting Cancer

### 3.1. YAP Action through the Stages of Cancer Development

**Figure 3:**
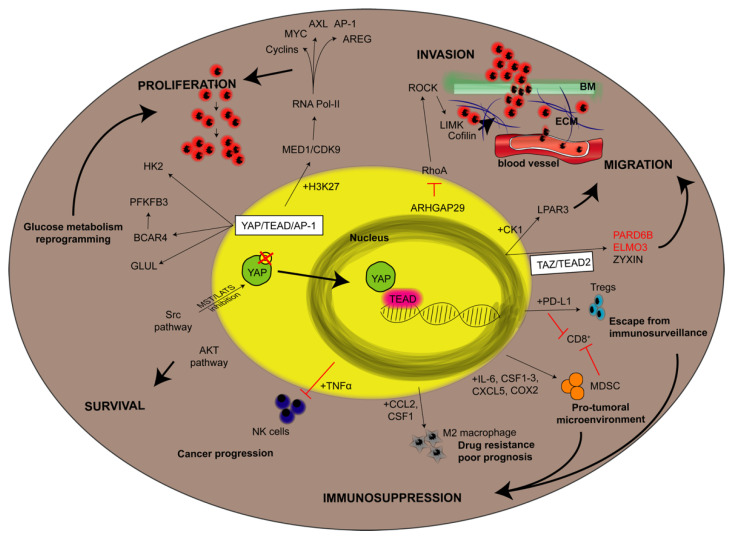
YAP downstream targets in cancer cells. The figure aims to give an overview about the downstream targets of YAP involved in a given step of cancer development taking into consideration studies led in vitro, in vivo and referring also to some correlations with poor prognosis in human patients. Steps of cancer development (indicated in bold uppercase) are proliferation, survival, invasion, migration and immunosuppression. They are linked to a given pathway by black bold arrows. Thin black arrows indicate either YAP-dependent protein overexpression (if the protein is reported in black) or protein downregulation (if reported in red). If the molecular pathway involves the contribution of additional YAP-dependent proteins expression, they are reported close to the arrows and preceded by “+”. Otherwise, red lines mean a block or an inhibition of a given pathway. AP-1: activator protein-1; AREG: amphiregulin; ARHGAP29: Rho GTPase activating protein 29; AXL: AXL receptor tyrosine kinase; BCAR4: breast cancer antiestrogen resistance 4; BM: basement membrane; CCL2: C-C motif chemokine ligand 2; CDK9: cyclin-dependent kinase 9; COX2: cyclooxygenase-2; CSF: colony stimulating factor; CXCL5: C-X-C motif chemokine ligand 5; ECM: extracellular matrix; ELMO3: engulfment and cell motility 3; GLUL: glutamate-ammonia ligase; H3K29: monomethylation oh histone 3; HK2: hexokinase 2; IL-6: interleukin 6; LIMK: LIM domain kinase 1; LY3009120: pan-RAF inhibitor; MED1: mediator complex subunit 1; MDSC: Myeloid-derived suppressor cells; NK: natural killer; LPAR3: LPA receptor 3; PARD6b: Par-6 Family cell polarity regulator beta; PD-L1: programmed death-ligand 1; PFKFB3: phosphofructokinase; ROCK: Rho-associated coiled-coil containing protein kinase 1; Tregs: regulatory T cells; 5FU: 5 fluorouracil.

Almost all cellular signals that have YAP as a main downstream target effector (i.e., mechanotransduction, Rho GTPase, Wnt signaling, inflammation and metabolic variation [77,81,85,86,87,88,89,90]) (Figure 3) are highly involved in the regulation of tissue repair, and by referring to cancers as “wounds that do not heal”, the prominent involvement of YAP in the regulation of tumor growth appears logical [91]. Accordingly, sustained cell proliferation is among the best characterized YAP-driven biological responses, emphasizing its remarkable involvement in tissue development by directly acting on the amplification of progenitor cells.

#### 3.1.1. Proliferation

Systematically, YAP controls cell proliferation by regulating the expression of genes directly involved in the cell cycle such as cyclins, mitotic kinases, proteins involved in DNA replication and repair, and transcriptional regulators [92,93,94,95]. Notably, all previous studies aiming to elucidate the regulatory role of YAP in cell proliferation have been conducted in tumoral environments, where YAP is highly active. Accordingly, abundant literature has shown abnormal YAP activity in virtually every cancer-associated process by both in vitro and in vivo analysis [96].

One of the first studies correlating alterations in YAP to cancer development showed that YAP was present in the 350-kilobase amplicon found in a mouse mammary tumor and that the overexpression of human YAP promoted EMT, inhibited apoptosis and enhanced growth factor-independent proliferation in mammalian epithelial cells [97]. Subsequently, YAP was also identified on the 11q21 human amplicon, which is amplified in numerous cancer types [98,99,100]. Finally, to corroborate its role in cancer development, genomic analysis revealed that YAP is mutationally activated in different cancer types, such as hemangioendothelioma, ependymoma, poroma, porocarcinoma and mesothelioma [101,102,103]. Despite those somatic mutations, the connection between YAP overexpression and tumor growth has been reported by countless studies analyzing most solid tumors, such as lung, colorectal, breast, pancreatic, and liver carcinomas, as well as melanoma and glioma. In these last tumor types, where a genetic alteration of YAP is not present, its role in promoting tumor development is associated with Virchow’s cancer theory, in which sustained inflammation and aberrant tissue regeneration are the crucial drivers of tumorigenesis [91,104].

For all the cancer types listed, scientists have conducted in vitro analyses on immortalized tumoral cell lines and in vivo studies by injecting syngeneic tumoral cells into mouse models to confirm the active role of YAP in tumor growth, migration and metastasis [104]. As extensively summarized by Thomson, both in vitro and in vivo studies were conducted on colorectal, breast, pancreatic and lung adenocarcinoma, squamous cell and hepatocellular carcinomas, cholangiocarcinoma, melanoma, gastric and brain cancers, showing that the downregulation of YAP results in reduced mass formation, cancer cell dissemination (either invasion and migration) and metastasis development [104]. Of course, oncogenic YAP hyperactivation depends also on all those dysregulated upstream regulators which have been listed in chapter 2 and that are peculiar for the environment of a given cancer. A careful analysis conducted in solid tumors (i.e., lung cancers [105]) highlighted a role in the YAP-mediated tumorigenesis and metastasis to the downregulation of LATS1/2 and AMOT proteins, to the aberrant expression of ABL kinases, the inactivation of either liver kinase B1 (LKB1), identified to suppress YAP action [106], or Ski, whose promoter becomes hypermethylated [107].

The inappropriate activation of YAP in virtually every cancer type is further supported by a large variety of studies analyzing the direct correlation between YAP expression within cancer cells and patient prognosis [96,108]. A systematic meta-analysis of the literature combining 21 different studies with a total of 2983 patients demonstrated that both nuclear and total YAP protein overexpression were directly associated with reduced overall survival and adverse disease-free survival times [109].

As previously suggested, the protumorigenic role of YAP is dependent on its nuclear translocation and its association with TEAD 1-4, which allows its binding with DNA [110,111], even if most associations with chromatin targets require DNA looping and the involvement of enhancers [108]. In fact, ChIP-seq analyses revealed that the YAP transcriptional response is mediated by two elements, namely, TEAD factors and activator protein-1 (AP-1, dimer of JUN and FOS proteins), which allow YAP/DNA interaction at cis-regulatory elements containing both TEAD and AP-1 motifs. Once bound, the YAP/TEAD/AP-1 complex regulates gene transcription, activating different mechanisms: it promotes p300-dependent acetylation at lysine 27 of histone H3 [111], and it recruits the Mediator complex and induces transcriptional elongation by RNA Pol-II [110]. These processes promote the synergic activation of a large variety of cell cycle regulators such as genes controlling S-phase entry (such as Cyclin D, AXL and FOXM1), mitosis (such as AREG) and DNA duplication and DNA repair [94,112,113,114]. Moreover, YAP and TAZ are able to self-sustain their expression by triggering a positive feedback loop that induces the expression of their genes and by activating and potentiating the expression of other proto-oncogenes, such as MYC and AP-1 family factors [115,116,117].

YAP-induced tumor growth is highly enhanced by AP-1 overexpression, but AP-1 proliferation-enhancing activity is inefficient in the absence of YAP and TAZ. Other ligands involved in the regulation of tissue growth, such as proteins of the Wnt, Notch, EGF (see above), bone morphogenetic protein (BMP)/TGF-β and JAK-STAT signaling pathways, have been shown to be targeted by YAP [118].

With the concomitant transcriptional activity in favor of the expression of pro-survival genes, YAP is able also to mediate genetic repression of several tumor suppressor genes. This topic has been reported very recently in two seminal papers by Hoxha et al. [119] and Lo Sardo et al. [120]. The first describes how a molecular complex composed by the association of YAP with the factor Yin Yang 1 (YY1), Polycomb repressive complex (PRC2) and the trimethylation of Histone H3 protein (which means genetic silencing) can inhibit CDKN1B gene [119]. Encoding for p27, a protein essential to limit S phase entry of the cell cycle, this clearly sustains cell proliferation. The second one demonstrated once again how the TGF-β pathway and YAP signaling are interconnected. Indeed, YAP expression induces a significant downregulation of TGF-β Receptor 2 (TGFBR2) through the orchestrated action of miR-106b-25, post-transcriptionally, and with the help of Enhancer Of Zeste 2 (EZH2, the functional active part of PRC2), a new YAP target involved in the triple methylation of Histone H3 [120]. EZH2 would act as mediator, without any kind of heterodimerization with YAP, but the depletion of both proteins reduces colony formation and tumor growth [120].

Carcinogenesis requires not only extensive cellular proliferation but also adequate metabolic support to sustain cell maintenance [121]. Interestingly, YAP is also involved in the high metabolic requirements of tumor cells through the upregulation of enzymes involved in glutamine metabolism, the hexosamine biosynthesis pathway, nucleotide biosynthesis and glycolysis [122,123,124,125]. Moreover, YAP stimulates BCAR4 lncRNA expression, upregulating glycolytic factor 6-phosphofructo-2-kinase/fructose-2,6-biphosphatase 3 (PFKFB3) [126] and thus supporting aerobiotic glycolytic-mediated YAP/TAZ transcriptional responses [77].

#### 3.1.2. Invasion and Migration

Another important trait of tumor cells is their ability to invade the extracellular matrix. Notably, among YAP targets, there are many genes involved in cell adhesion, migration and angiogenesis such as connective tissue growth factor (CTGF), cysteine-rich angiogenic inducer (CYR61) and integrins [127,128]. Moreover, the migratory process is sustained by the cooperation of YAP and TAZ with myocardin-related transcription factor (MRTF) and serum response factor (SRF) for the activation of cancer-associated fibroblasts, matrix stiffening, and metastasis [104]. In the tumorigenic process, cell migration is preceded by the detachment of the cell from its surrounding environment and the initiation of EMT. Interestingly, YAP represses the programmed cell death prompted by the loss of attachment to the extracellular matrix (anoikis) [129], which is one of the first steps of EMT. Additionally, TAZ and YAP activation confers cancer stem cell traits to immortalized breast cancer cells [130], thus mimicking the staminal induction that is endowed by EMT [131]. However, the correlation between EMT and YAP expression remains to be fully elucidated. Some studies demonstrate that YAP overexpression supports EMT [132] and that, vice versa, EMT induces and requires YAP and TAZ expression [130], while EMT is not mandatory for YAP-induced cell plasticity [133].

EMT represents the first cellular transformation that cancer cells undergo during the metastatic process, involving a continuous reorganization of the cytoskeleton of cancer cells, since they undergo constant shape changes required for migratory and invading progression. In this scenario, YAP supports cytoskeletal remodeling in cancer cells, not only acting as a mechanotransductor but also modulating different types of genes encoding proteins involved in cell migration, mainly in a cancer-type-specific way. The promotion of the metastatic process by YAP-TEAD interactions was first described by Lamar and colleagues in breast cancer and melanoma cells [134]. In gastric cancer, YAP was found to regulate F-actin dynamics through the transcription of genes encoding Rho GTPase-activating protein 29 (ARHGAP29) [135,136]. In pancreatic cancer, YAP promotes cell motility and invasion via LPA receptor 3 (LPAR3) [136], while in mesothelioma and breast cancer cells, YAP mediates the expression of receptor of hyaluronan-mediated motility (RHAMM). Finally, in breast cancer, YAP promotes migration and invasion by promoting ZYNX [137] (Figure 2). These experimental data are corroborated by several clinical studies demonstrating the correlation between YAP or TAZ overexpression and the invasiveness of human cancers [138,139,140,141,142].

#### 3.1.3. YAP as Tumor Suppressor

Additionally, some studies have reported that YAP expression has a negative effect on the metastatic process, even repressing it, thus resulting in a more positive prognosis [109,143,144,145]. Similarly, some findings emphasized a YAP-driven tumor suppressive response. YAP overexpression in hepatocytes does not lead to cellular clonal expansion unless the target cell receives a second protumorigenic signal, such as tissue damage or inflammation, from the surrounding microenvironment [146]. In the absence of these latter signals, YAP overexpression is counterbalanced by increased apoptosis [147]. Analogous tumor suppressive responses have also been reported to exist in colorectal cancer [148].

However, it is in the hematological malignancies that the YAP tumor suppressor activity has been widely described [149]. Despite these kinds of cancer display DNA damage and an attempt to initiate a regulated cell death mechanism, these cells finally escape from apoptosis, demonstrated to be caused by low levels of YAP [150]. It has been shown that forced nuclear accumulation of YAP following the inhibition of MST1/2 kinases restored cell death in multiple myeloma (MM). With the same rationale but different target, Maruyama J. et al. identified a novel activator of YAP, enriching possibilities to pharmacologically treating MM. This, named as IBS003031, increases the amount of the unphosphorylated protein consequent of increased interactions with phosphatases and through unknown mechanisms which, in any case, lead to death of the transformed cells [151]. Again, one of the Hippo pathway components and main regulator of YAP degradation via the phosphorylation cascade LATS1/2 kinases-mediated, is MOB1. MOB1 was found increased in MM in a process dependent from PINK1-mediated mitophagy [152]. The significant increased instability of YAP and the unedited role of mitophagy shed new lights on the pathogenesis of MM.

These findings might suggest that YAP activation plays a variable role in the tumorigenic process: on the one hand, YAP might be responsible for triggering the event, but YAP also requires external signals from the surrounding microenvironment to sustain the process, further supporting Virchow’s cancer theory. YAP interactions with the surrounding tumoral microenvironment and the role of YAP in chemoresistance are further discussed in the chapter below.

### 3.2. YAP at the Intersection of Immune and Tumor Cells

As introduced in the previous paragraph, the microenvironment surrounding cancer cells plays a pivotal role in the progression of the tumorigenic process. Inflammation and tissue damage are responsible for the activation of a variety of signaling cascades, thus generating an ideal environment promoting tumor growth. In this scenario, a crucial part is innate immunity, represented mainly by tumor-infiltrating lymphocytes (TILs) and tumor-associated macrophages (TAMs). YAP expression, like the whole Hippo pathway, plays an important role in the regulation of the biological activity of immune cells, including development, proliferation, differentiation and function [153]. Even if YAP activity is fundamental for the correct development of immune cells and, therefore, its abnormal expression could also affect tumor growth, in this section, we will focus only on the role of tumoral YAP expression in the regulation of cancer immunity.

The tumor microenvironment (TME) deeply influences cancer development through the immunomodulatory effect exerted by the expression of YAP and TAZ. Specifically, the expression of YAP and TAZ by tumor cells has a direct effect on TILs, TAMs, and myeloid-derived suppressor cells (MDSCs) and regulates the expression of programmed death ligand 1 (PD-L1; also known as CD274), which binds to PD-1, a type I transmembrane protein expressed on activated T cells, B cells, monocytes, NK cells, and DCs [154] (Figure 2).

#### 3.2.1. TILs and MDSCs

One of the main types of TILs is T cells. Among the T cells, CD8^+^ cells are responsible for the immune surveillance of tumors, while CD4^+^CD25^+^ regulatory T cells (Tregs) promote tumor progression by suppressing effector T cell activity [155,156,157]. Interestingly, immunohistochemical analysis of several gastric adenocarcinoma tissues revealed a positive correlation between the expression of YAP in tumoral cells and the number of infiltrating Tregs [158]. In addition, in Kras:Trp53-mutant pancreatic ductal cancer cells (PDAC), YAP expression has been observed to prevent the activation of CD8^+^ cells [159]. In both of the described studies, YAP overexpression in tumor cells negatively effects the innate immune response against cancer growth, thus favoring the tumorigenic process. Conversely, Moroishi and collaborators demonstrated that LATS1-2-deficient tumor cells showed reduced in vivo tumor growth, while in vitro YAP/TAZ hyperactivation promoted tumoral expansion. This mechanism was entirely due to the interaction between the tumor cells and the TME: LATS1-2-null tumor cells induce type I IFN signaling by releasing nucleic acid–rich extracellular vesicles, thus facilitating CD8^+^ T cell expansion and tumor suppression [160]. This latter study highlights how YAP hyperactivation suppresses tumor growth in vivo via a transcription-dependent mechanism and that inactivation of the Hippo pathway in tumor cells stimulates host immune responses. These findings suggest the existence of a complex mechanism beyond the interaction between tumoral YAP and TME regulation, which still needs to be fully elucidated.

The communication between cancer cells and immune cells in the TME is mediated by the release of specific chemokines and cytokines that are able to activate different responses in the target cells. Interestingly, in the same Kras:p53-mutant PDAC model cited below, YAP expression was shown to be responsible for the production and secretion of interleukin 6 (IL-6) and macrophage colon-stimulating factor 1-3 (CSF1-3), which are responsible for the recruitment and differentiation of MDSCs [159]. Briefly, MDSCs are a heterogeneous population of immature immune cells belonging to the myeloid lineage. Upon stimulation, MDSCs can differentiate into dendritic cells (DCs), macrophages and neutrophils and are known to be involved in the tumorigenic process of different types of cancers [161,162,163]. MDSCs are recruited by cancer cells and are responsible for generating a protumoral microenvironment, suppressing the antitumoral immune response by directly inhibiting CD8^+^ cytotoxic T cells [164]. Consequently, differentiated MDSCs lead to impaired T cell activation, resulting in poor patient prognosis. Interestingly, knockout of YAP in the Kras:p53-mutant PDAC model resulted in a reduction in MDSC infiltration with the consequent reactivation of CD8^+^ cytotoxic T lymphocytes and the apoptosis of neoplastic ductal cells [159]. Similar to pancreatic carcinoma, in prostate cancer, YAP activation also promotes the secretion of a protumorigenic chemokine, C-X-C motif chemokine ligand 5 (CXCL5), which is responsible for Cxcl5–Cxcr2-mediated MDSC recruitment, thus impeding T cell proliferation [165]. Moreover, YAP-driven MDSC activation has also been shown in ovarian carcinoma. In this latter scenario, immune suppression is mediated by the activation of protein kinase C iota (PRKCI) [166], which, interestingly, improves the nuclear localization of YAP, thus prompting the release of TNFα proinflammatory cytokines and the recruitment of MDSCs [167,168]. Last, in colorectal cancer, YAP expression has been shown to directly correlate with an increased density of MDSCs within the TME and, therefore, with more aggressive tumoral clinical features [169]. Mechanistically, in all the tumoral contests described, tumoral YAP together with TEAD stimulates the recruitment of MDSCs within the TME by promoting the production of different cytokines, such as IL-6, CSF1, CSF2, CSF3 and CXCL5. This mechanism highlights the multifaceted effects of YAP in the tumoral context, indicating its ability to act as a chemoattractant protumorigenic cells (Figure 3).

Another ability of YAP is to regulate the expression of PD-L1 in tumor cells, thus further affecting tumoral immunity. In fact, PD-L1 is expressed by a variety of tumor and immune cells [170], and its receptor, PD-1, is present on activated T cells, B cells, monocytes, NK cells, and DCs. Interestingly, PD-1/PD-L1 represent a fundamental immune checkpoint pathway in the TME and are responsible for the induction and preservation of T cell tolerance, thus maintaining physiological immune responses and avoiding undesired tissue injury [171]. For these reasons, any alteration of this immune checkpoint in a tumoral context, such as the expression of PD-L1 by cancer cells results in the impairment of antitumor immunity [172,173]. Different studies have recently reported that YAP regulates the expression of PD-L1 in melanoma, lung and mesothelioma cells [174,175,176]. In all the cancer types cited, YAP-expressing tumor cells showed the ability to evade the CD8^+^ T cell immune response in a PD-L1-dependent manner. YAP has been shown to regulate PD-L1 by binding its enhancer region [175,177,178]. Interestingly, in vitro treatment with PD-1 inhibitors can restore T cell effector functions, leading to the elimination of YAP-expressing melanoma cells [175]. However, a direct connection between PD-L1 and YAP expression in human tumors is still missing, since only limited data demonstrate a trend of low PD-L1 expression in tumors with low or no YAP expression, while the opposite has not been confirmed (high PD-L1 and high YAP expression) [179].

#### 3.2.2. TAMs

The most represented class of immune cells (in number and involved in inflammation) in TME is TAMs. Indeed, activated macrophages represent the most abundant tumor-infiltrating cell types and can be divided into two subgroups, namely, protumoral macrophages (M2) and antitumoral macrophages (M1), capable of differentially influencing the tumorigenic process and antitumoral treatments [180]. In most cases, TAMs behave like M2 macrophages, promoting cancer development, invasion and metastasis, and their presence is often associated with drug resistance and poor prognosis [181,182]. Interestingly, different studies have reported an association between YAP activation and macrophage recruitment in different mouse models of hepatocellular carcinoma (HCC) [183,184,185]. Notably, Guo and colleagues showed that the activation of YAP in a single hepatocyte of a healthy mouse is sufficient to recruit macrophages by enhancing C-C motif chemokine ligand 2/colony-stimulating factor 1 (Ccl2/Csf1) secretion, thus triggering escape from immune surveillance [185]. Moreover, YAP also induces the expression of monocyte chemoattractant protein-1 (Mcp1), further contributing to macrophage infiltration [186]. This tumoral YAP-TAMs connection might be extended to other malignancies; in colorectal cancer, YAP expression promotes M2 polarization, thus promoting protumoral effects of TAMs [187], while in a mouse model of pancreatic adenocarcinoma, YAP expression is responsible for TAMs recruitment [159]. Interestingly, the anti-inflammatory and antitumoral drug ovatodiolide has been shown to reduce YAP expression in colorectal cancer colon spheres, which is associated with the reduction in M2 TAMs polarization [187], thus emphasizing that YAP is a possible therapeutic target to combat the protumoral inflammatory response.

In conclusion, due to the important role of YAP in the regulation of the TME immune response (Figure 2), further studies on the development of specific therapies to modulate YAP-mediated protumoral effects might represent a promising adjuvant therapy for different types of cancer.

## 4. Biochemistry and Translational Properties of Currently Known YAP Inhibitors

A growing body of literature indicates that YAP signaling is associated with poor prognosis and is involved in the development of resistance to various chemotherapies, radiotherapies or immunotherapies. YAP activation is a major mechanism of resistance to multiple targeted therapies and helps tumor cells activate alternative survival pathways, bypass pathway inhibition and avoid apoptotic signaling [188]. Identification of the mechanisms by which YAP enables tumor cells to overcome the detrimental effects of chemotherapy drugs and to design optimal strategies to counteract relapse is thus essential. In the following paragraphs, we focus on ongoing research progress on drugs and small molecules that target YAP in different ways (Table 1). They will be subdivided in: drugs affecting nuclear localization of YAP, inhibitors which compete with YAP binding to molecular partners and other mechanisms of action. It is reported that each drug may have multiple routes of action, thus each of them will be included in the section for which several evidence are provided.

### 4.1. Drugs Affecting Nuclear Localization of YAP

One of the common strategies used by new drugs and small molecule inhibitors to counteract YAP function is linked to the inhibition of its nuclear localization. For example, A35, a novel synthetic cyclizing berberine, has been patented as an antitumoral compound that decreases YAP nuclear localization by activating its phosphorylation, which subsequently reduces the expression of its target genes [189]. Interestingly, dichloroacetate promotes the nuclear-cytoplasmic translocation of YAP, but not TAZ, which is essential for DCA-mediated effects on hepatocellular carcinoma cell stemness [190]. Among the small molecule inhibitors and drugs that target the YAP protein, norcantharidin (NCTD), a derivative of cantharidin, seems to specifically target the YAP signaling pathway, modulating the subcellular distribution of YAP between the nucleus and cytoplasm; however, these data have been retracted from the publication due to lack of overall confidence [191].

Interestingly, pazopanib, together with statins and dasatinib, inhibits YAP/TAZ nuclear localization and target gene transcription and induces YAP/TAZ phosphorylation; a combination of these compounds with each other or with other anticancer treatments, such as doxorubicin or paclitaxel, efficiently reduces the proliferation of YAP/TAZ-dependent breast cancer cells [192].

### 4.2. Competitive Inhibition of YAP Binding and Function

YAP upregulation is a mediator of acquired and intrinsic resistance in KRAS-mutant colon [193] and lung cancer cells [194]; interestingly, its inhibition is sufficient to sensitize these cells to pharmacological treatment (i.e., selumetinib and momelotinib) and to prevent them from acquiring resistance [194].

Verteporfin (VP), a benzoporphyrin derivative used as a photosensitizer in photodynamic therapy, was the first compound identified to directly inhibit YAP by either disrupting YAP-TEAD interactions and preventing YAP-induced tumorigenesis [195,196] or by suppressing the expression of YAP/TAZ transcriptional targets, including EGFR, conferring survival benefits in a glioblastoma model [197] (Table 1). VP has also been shown to strongly inhibit the migration of different molecular subtypes of breast cancer cells [198] and to induce significant apoptosis in osteosarcoma cells [199]. However, VP also has YAP-independent effects [200,201] and seems to be ineffective in vivo [202]; therefore, it is not a clinically viable YAP inhibitor.

Notably, BET family protein BRD4 inhibitors (BRD4-i) represent another potential approach to overcome YAP/TAZ-induced drug resistance by inhibiting YAP/TAZ protumorigenic activity both in vitro and in vivo [203]. JQ1 BRD4, belonging to BET protein family members, suppresses YAP and its transcriptional program and resensitizes lung cancer to chemotherapy treatment [194]. These results revealed that higher YAP expression contributes, at least partially, to intrinsic resistance to chemotherapy and that combination with JQ1 might be broadly effective.

A protein with inhibitory functions of the YAP-TEAD transcriptional complex is Vestigial Like Family Member 4 (VGLL4), which is a direct competitor for TEAD4 binding that promotes growth-dependent YAP, thus suppressing lung tumor cell growth [204] and regulating breast cancer pathogenesis. A peptide mimicking VGLL4 function, Super-TDU, has been synthesized and acts as an efficient YAP antagonist to inhibit gastric cancer growth both in vitro and in vivo [205]. The small molecule CA3 has been demonstrated to be an effective YAP inhibitor. CA3 inhibits YAP/TEAD transcriptional activity, induces apoptosis and synergistically potentiates the effect of 5-fluoracil in resistant esophageal adenocarcinoma cells with high YAP expression [206]. Moreover, CA3 exhibits potent inhibitory effects on oral squamous cell carcinoma (OSCC) migration [207] and reduces resistance to anoikis in melanoma cells [208].

Other and less known YAP/TEADs disruptors are reviewed in [31].

### 4.3. Other Inhibitors

Genetic or pharmacological inhibition of YAP (by the use of the previously mentioned VP) significantly enhances the antitumor efficacy of the pan-RAF inhibitor LY3009120 in KRAS-mutant pancreatic cancer by blocking compensatory AKT signaling pathway activation [209]. Statins, a class of drugs largely used in the treatment of hypercholesterolemia, block YAP/TAZ nuclear localization and activity through Rho-GTPases and have tumor-suppressor activities [81,210]. Moreover, combined EGFR and YAP inhibition (with statins) is an effective strategy to prolong survival in lung cancer patients [211]. However, statins also seem to be more efficacious in vitro than in vivo, suggesting that finding the right concentration for their efficacy against tumors is not easy.

A different approach for disrupting YAP activity is by indirectly regulating upstream factors, such as Src kinase. Inhibition of Src kinase via treatment with dasatinib, a thiazole carboximide derivative, decreases YAP transcriptional activity and target gene expression and impairs tumor growth and metastasis [47]. However, Src influences YAP and TAZ through multiple distinct mechanisms; thus, direct inhibition of Src itself rather than Src effector pathways may be a good strategy. In addition, dasatinib notably suppresses YAP activity via a Src-JNK-LIMD1-LATS-dependent pathway in renal cell carcinoma, and the alteration of YAP phosphorylation (S127) could serve as a biomarker of the response to dasatinib [212]. Recently, nuciferine, an alkaloid extracted from plants, attenuated gemcitabine resistance in pancreatic cancer cells, efficiently inhibiting YAP via AMPK-mediated downregulation [213].

Radiotherapy (RT) remains a crucial method for cancer treatment [214]. Despite recent advancements in radiation technologies and multidisciplinary approaches, different tumors display radioresistance. Several studies have reported that high levels of YAP predict a poor response to RT. YAP activation induces resistance to radiation, while silencing YAP increases sensitivity to RT and potentiates the DNA damage response [215,216,217]. Genetic YAP inhibition or treatment with VP sensitizes triple-negative breast cancer cells to RT by targeting the DNA damage response and cell survival pathways [218]. Interestingly, the C38 antibody, which targets glucose-related protein 78 kDa (GRP78), dramatically reduces the expression of YAP targets and enhances the efficacy of RT [219]. Taken together, these data indicate that YAP activation represents a pharmacological target to enhance the antitumor effects of DNA damage mechanisms and that YAP inhibitors in combination with RT treatment represent a promising strategy for the treatment of cancer.

Finally, there is some evidence that YAP confers resistance of cancer cells to immunotherapy [153]; thus, understanding its role in tumor immunity may be essential for exploring innovative tumor treatments that take advantage of YAP inhibition combined with immunotherapy.

Further studies are needed to determine the exact contributions of YAP to overall resistance development to make further breakthroughs in cancer treatment.

## 5. Conclusions

One fascinating matter in biology is how the Hippo (YAP) signaling pathway can transduce signals from a wide range of stimuli (physical, mechanical, chemical and biological) via proper and finely regulated responses. The responses are not confined to a given developmental stage or to a given physiological process but occur under multiple conditions at multiple levels, with feedback mechanisms and tissue specificity, to sustain each stage of organogenesis and cancer development. Increasing our understanding of this pathway by revealing new components and understanding all nonredundant roles of YAP and TAZ and how they are dysregulated in cancer may help not only to explain the most frequently observed effects that have been left unexplained for years but also to develop new strategies for the treatment of cancer.

## Figures and Tables

**Table 1:** Summary of the most known YAP inhibitors.

Drug	Mode of Action	Preclinical (P)/Clinical (C) Use
A35	Lower YAP nuclear accumulation	P
DCA	Lower YAP nuclear accumulation	P/C
NCTD	Lower YAP nuclear accumulation	P
Pazopanib	Lower YAP nuclear accumulation	P/C
Dasatinib	Lower YAP nuclear accumulation	P/C
	Blocking Src kinase signaling	
Statins	Lower YAP nuclear accumulation	P/C
VP	Competitive binding to YAP	P/C
BET inhibitors (JQ1)	Competitive binding to YAP	P/C
VGLL4	Competitive binding to YAP	P
Super TD4	Competitive binding to YAP	P
CA3	Competitive binding to YAP	P
VP+LY3009120	Blocking AKT signaling	P/C
Nuciferine	Blocking AMPK signaling	P
C38+RT	YAP downregulation	P/C

DCA: dichloroacetate; NCTD: norcantharidin; RT: radiotherapy; VGLL4: vestigial-like family member 4; VP: verteporfin.
